# Supplementary material for: A Comprehensive Study of the Synthesis, Spectral Characteristics, Quantum–Chemical Molecular Electron Density Theory, and In Silico Future Perspective of Novel CBr3-Functionalyzed Nitro-2-Isoxazolines Obtained via (3 + 2) Cycloaddition of (E)-3,3,3-Tribromo-1-Nitroprop-1-ene
Source: Molecules. 2025 May 13;30(10):2149. doi: 10.3390/molecules30102149 (PMC12114255; doi:10.3390/molecules30102149)
Supplement: Supplementary file 1 [file molecules-30-02149-s001.zip › molecules-3588968-supplementary.pdf]

SUPPLEMENTARY MATERIALS

A Comprehensive Study of the Synthesis, Spectral Characteris-tics, Quantum–Chemical Molecular Electron Density Theory, and In Silico Future Perspective of Novel CBr3-Functionalyzed Nitro-2-Isoxazolines Obtained via (3 + 2) Cycloaddition of (E)-3,3,3-Tribromo-1-Nitroprop-1-ene

Karolina Zawadziska-Wrochniak 1, Karolina Kula 1,*, Mar Ríos-Gutiérrez 2, Bartomiej Gostyski 3, Tomasz Krawczyk 4 and Radomir Jasiski 1,*

1 Department of Organic Chemistry and Technology, Cracow University of Technology, Warszawska 24, 31-155 Cracow, Poland

2 Department of Organic Chemistry, University of Valencia, Dr. Moliner 50, Burjassot, 46-100 Valencia, Spain

3 Department of Structural Chemistry, Centre of Molecular and Macromolecular Studies Polish Academy of Sciences, Sienkiewicza 112, 90-363 Łódź, Poland

4 Department of Chemical Organic Technology and Petrochemistry, Silesian University of Technology, Krzywoustego 4, 44-100 Gliwice, Poland

* Correspondence: karolina.kula@pk.edu.pl (K.K.); radomir.jasinski@pk.edu.pl (R.J.)

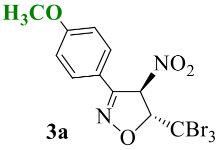

(4*RS*,5*RS*)-3-(4-methoxyphenyl)-4-nitro-5-tribromomethyl-2-isoxazoline (**3a**): isolation and purification process: column chromatography (Hex : EtOAc 9 : 1 *v/v*) and future crystallization (Et_2O : CyP 5 : 5 *v/v* on cold) m.p. 112.4 °C (white crystal solid)

R_f = 0.59; UV-Vis (MeOH): λmax [nm] 268; FT-IR (ATR): υ [cm^{-1}] 1606 (>C=N− 2-isox. ring), 1580 asym. and 1365 sym. (−NO_2), 1258 (~C-O-N= 2-isox. ring), 821 (~C-Br); ^{1}H NMR (400 MHz, $CDCl_3$): δ [ppm] 7.66-7.62 (m, 4H); 6.28 (d, 1H, J = 3.91 Hz); 5.86 (d, 1H, J = 3.92 Hz); 3.86 (s, 3H); ^{13}C NMR (100 MHz, $CDCl_3$): δ [ppm] 162.4; 162.3; 151.1; 128.7; 126.9; 112.2; 95.7; 93.9; 55.5; HR-MS (ESI−): calculated for $C_{11}H_9N_2O_4Br_3$ $[M-H]^-$ = 468.8034, found = 468.8055

HR-MS

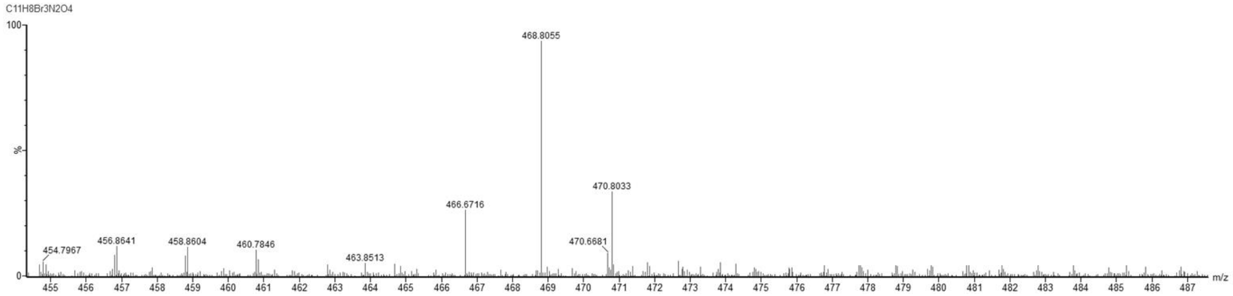

Figure S1. HR-MS spectrum of (4*RS*,5*RS*)-3-(4-methoxyphenyl)-4-nitro-5-tribromomethyl-2-isoxazoline (**3a**).

^{1}H NMR

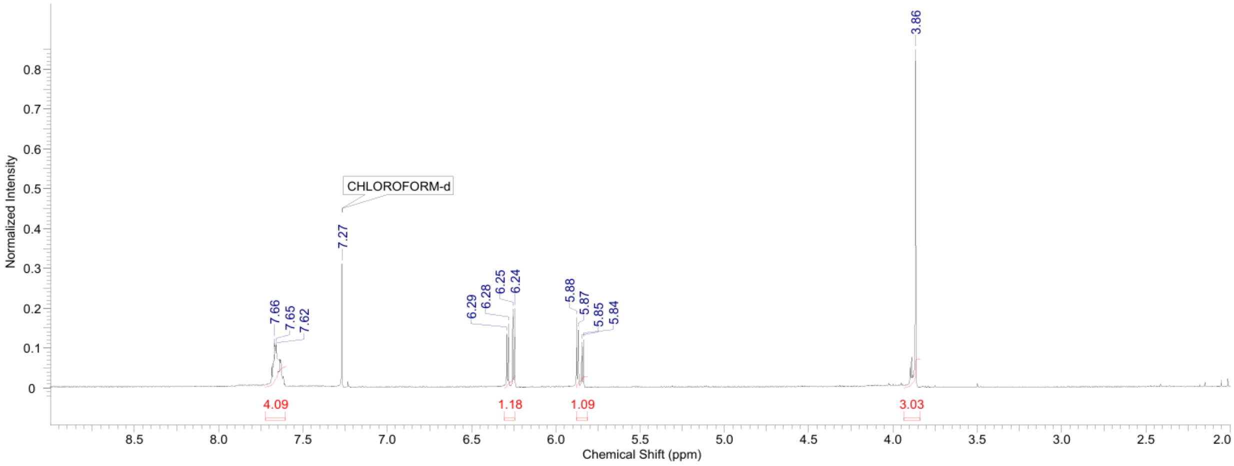

Figure S2. ^{1}H NMR spectrum of (4*RS*,5*RS*)-3-(4-methoxyphenyl)-4-nitro-5-tribromomethyl-2-isoxazoline (**3a**).

^{13}C NMR

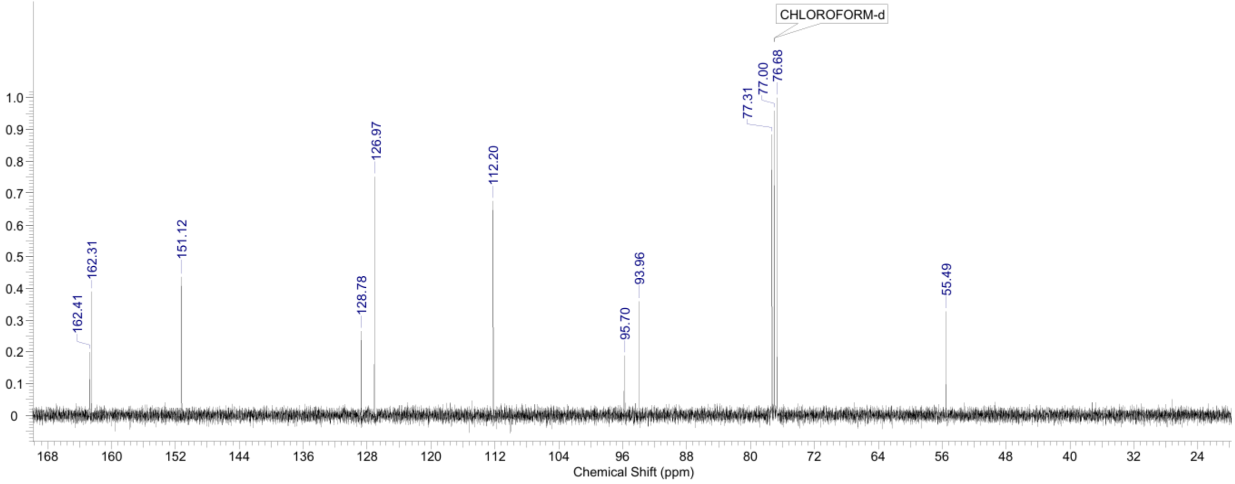

Figure S3. ^{13}C NMR spectrum of (4*RS*,5*RS*)-3-(4-methoxyphenyl)-4-nitro-5-tribromomethyl-2-isoxazoline (**3a**).

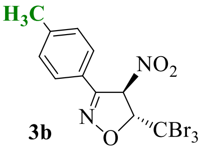

3b

(4*RS*,5*RS*)-3-(4-methylphenyl)-4-nitro-5-tribromomethyl-2-isoxazoline (**3b**): isolation and purification process: column chromatography (Hex : EtOAc 9 : 1 *v*/*v*) and future crystallization from ethanol
m.p. 86.5 °C (white crystal solid)

R_f = 0.47; UV-Vis (MeOH): λmax [nm] 265; FT-IR (ATR): υ [cm^{-1}] 1605 (>C=N− 2-isox. ring), 1578 asym. and 1366 sym. (−NO_2), 1259 (~C-O-N= 2-isox. ring), 819 (~C-Br); ^{1}H NMR (400 MHz, $CDCl_3$): δ [ppm] 7.77-7.72 (m, 4H); 6.30 (d, 1H, J = 4.21 Hz); 5.85 (d, 1H, J = 4.22 Hz); 2.41 (s, 3H); ^{13}C NMR (100 MHz, $CDCl_3$): δ [ppm] 154.6; 151.5; 142.5; 130.2; 127.0; 122.4; 95.7; 70.2; 21.5; HR-MS (ESI−): calculated for $C_{11}H_9N_2O_3Br_3$ [M-H]$^-$ = 452.8085, found 452.8108

HR-MS

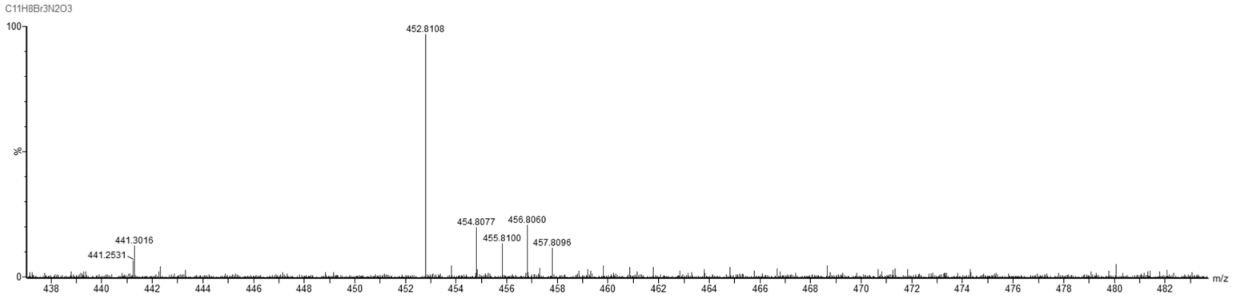

Figure S4. HR-MS spectrum of (4*RS*,5*RS*)-3-(4-methylphenyl)-4-nitro-5-tribromomethyl-2-isoxazoline (**3b**).

^{1}H NMR

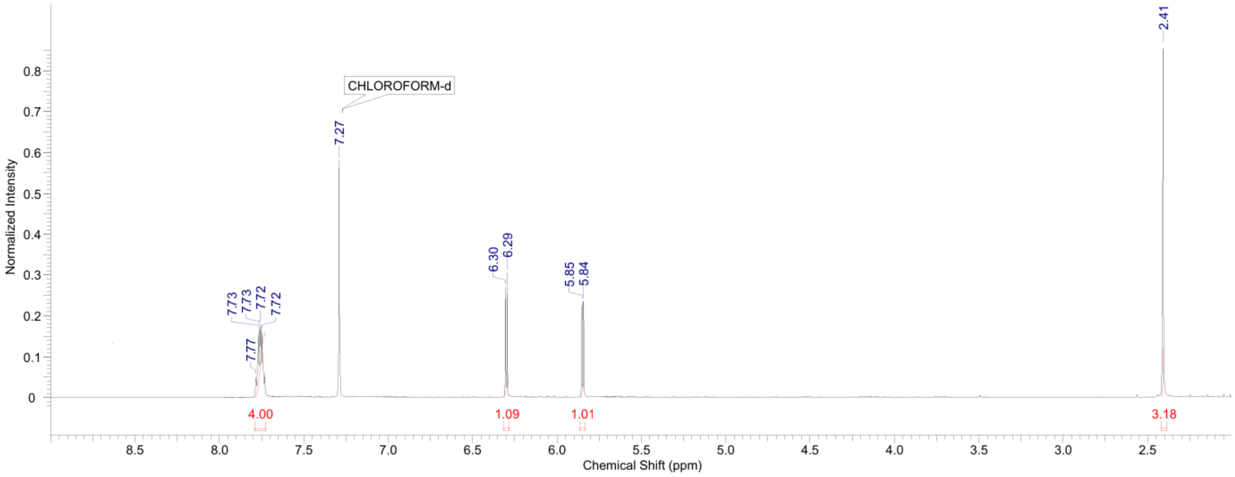

Figure S5. ^{1}H NMR spectrum of (4*RS*,5*RS*)-3-(4-methylphenyl)-4-nitro-5-tribromomethyl-2-isoxazoline (**3b**).

^{13}C NMR

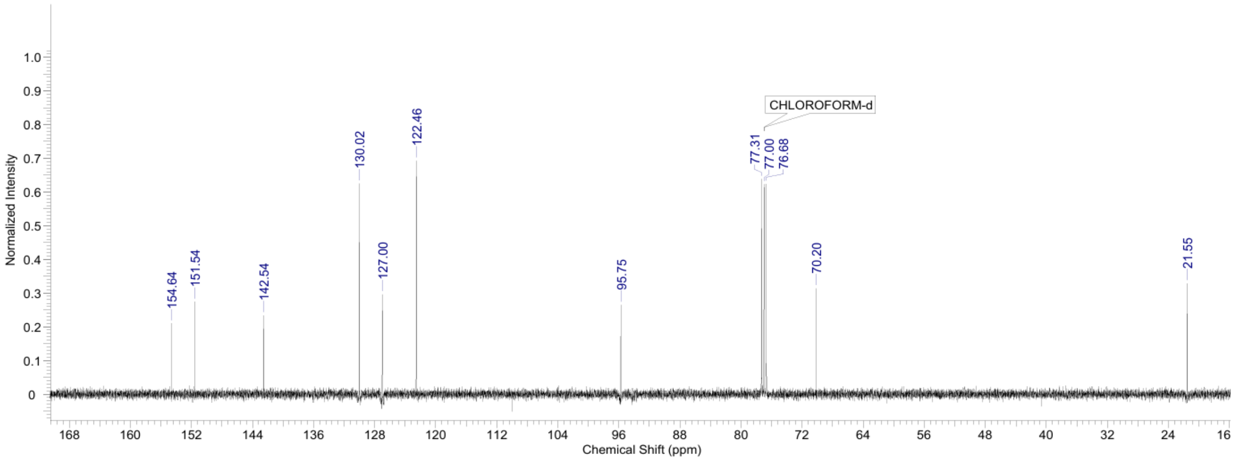

Figure S6. ^{13}C NMR spectrum of (4*RS*,5*RS*)-3-(4-methylphenyl)-4-nitro-5-tribromomethyl-2-isoxazoline (**3b**).

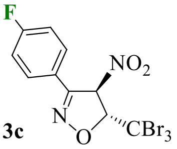

3c

(4*RS*,5*RS*)-3-(4-fluorophenyl)-4-nitro-5-tribromomethyl-2-isoxazoline (**3c**): isolation and purification process: column chromatography (Hex : EtOAc 9 : 1 *v*/*v*) and future crystallization from methanol m.p. 111.3 °C (white crystal solid)

R_f = 0.46; UV-Vis (MeOH): λmax [nm] 258; FT-IR (ATR): υ [cm^{-1}] 1601 (>C=N− 2-isox. ring), 1566 asym. and 1361 sym. (−NO_2), 1249 (~C-O-N= 2-isox. ring), 1231 (~Ar-F), 817 (~C-Br); ^{1}H NMR (400 MHz, $CDCl_3$): δ [ppm] 7.82-7.78 (m, 4H); 6.29 (d, 1H, J = 3.95 Hz); 5.90 (d, 1H, J = 3.96 Hz); ^{13}C NMR (100 MHz, $CDCl_3$): δ [ppm] 165.9; 163.4; 150.6; 129.2; 121.6; 116.8; 95.9; 94.0; HR-MS (ESI−): calculated for $C_{10}H_6N_2O_3Br_3F$ $[M{-}H]^-$ = 456.7834, found = 456.7850

HR-MS

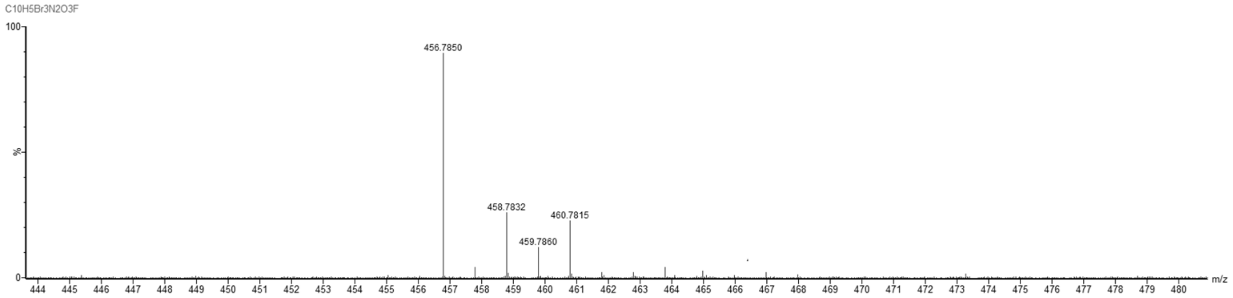

Figure S7. HR-MS spectrum of (4*RS*,5*RS*)-3-(4-fluorophenyl)-4-nitro-5-tribromomethyl-2-isoxazoline (**3c**).

^{1}H NMR

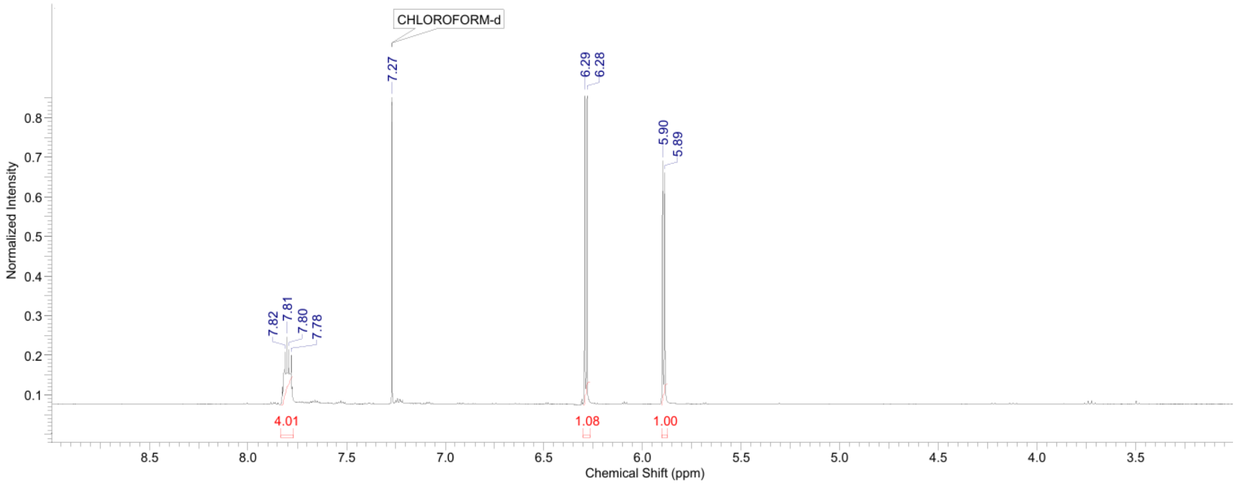

Figure S8. ^{1}H NMR spectrum of (4*RS*,5*RS*)-3-(4-fluorophenyl)-4-nitro-5-tribromomethyl-2-isoxazoline (**3c**).

^{13}C NMR

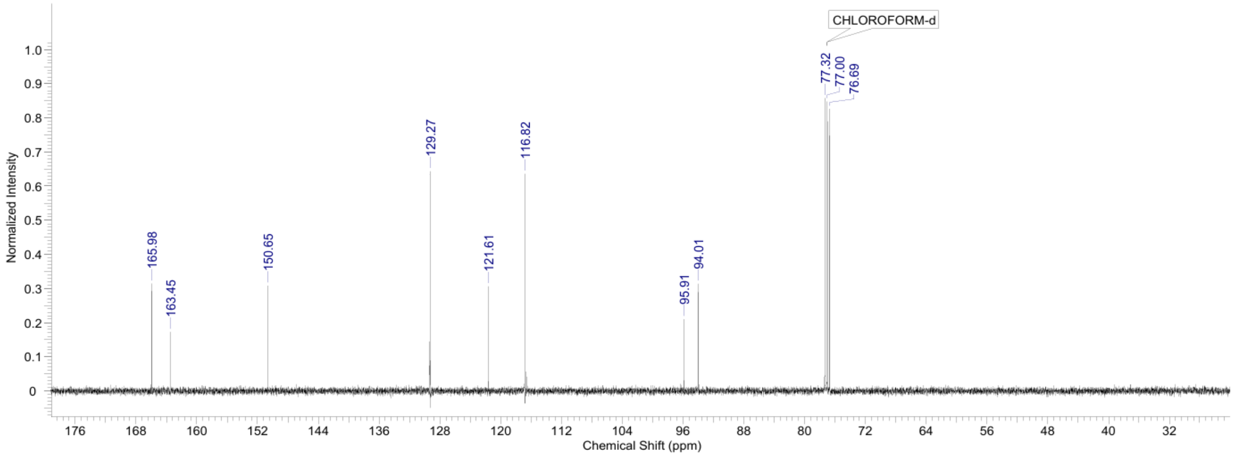

Figure S9. ^{13}C NMR spectrum of (4*RS*,5*RS*)-3-(4-fluorophenyl)-4-nitro-5-tribromomethyl-2-isoxazoline (**3c**).

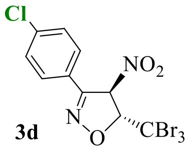

3d

(4*RS*,5*RS*)-3-(4-chlorophenyl)-4-nitro-5-tribromomethyl-2-isoxazoline (**3d**): isolation and purification process: column chromatography from chloroform and future crystallization from petroleum ether on cold greenish-yellowish semi-solid

R_f = 0.51; UV-Vis (MeOH): λmax [nm] 262; FT-IR (ATR): υ [cm^{-1}] 1591 (>C=N− 2-isox. ring), 1557 asym. and 1354 sym. (−NO_2), 1252 (~C-O-N= 2-isox. ring), 819 (~C-Br), 556 (~Ar-Cl); ^{1}H NMR (400 MHz, $CDCl_3$): δ [ppm] 7.76-7.70 (m, 4H); 6.27 (d, 1H, J = 3.87 Hz); 5.91 (d, 1H, J = 3.86 Hz); ^{13}C NMR (100 MHz, $CDCl_3$): δ [ppm] 168.0; 167.5; 153.0; 129.7; 125.1; 117.4; 95.9; 93.5; HR-MS (ESI−): calculated for $C_{10}H_6N_2O_3Br_3Cl$ $[M−H]^−$ = 472.7539, found = 472.7547

HR-MS

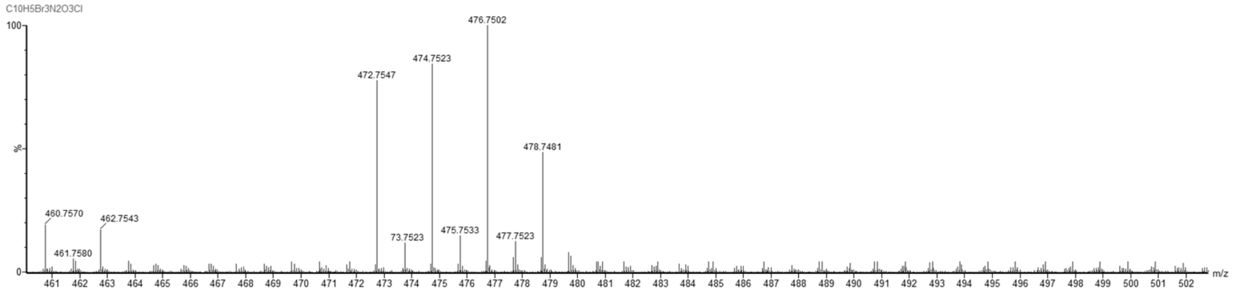

Figure S10. HR-MS spectrum of (4*RS*,5*RS*)-3-(4-chlorophenyl)-4-nitro-5-tribromomethyl-2-isoxazoline (**3d**).

^{1}H NMR

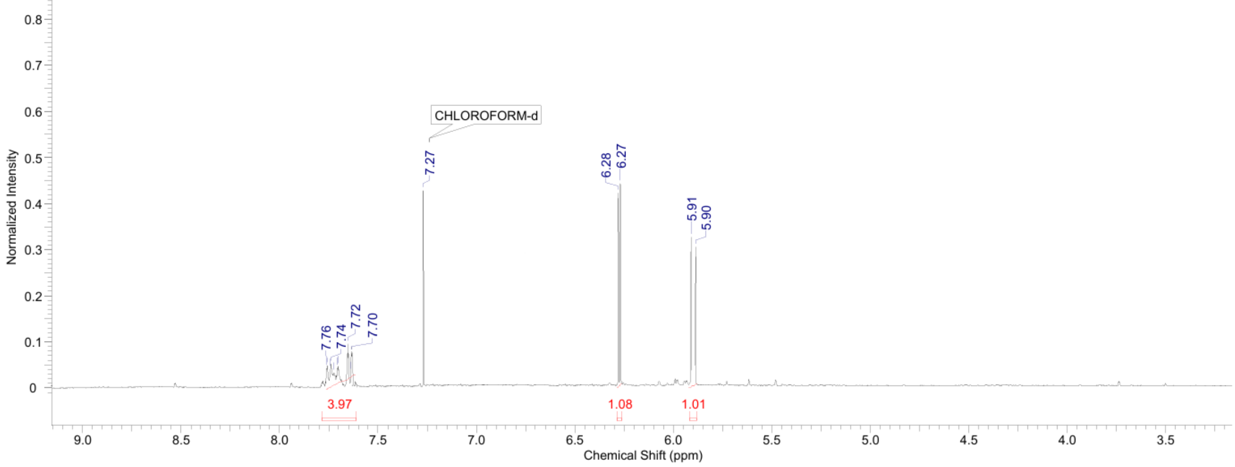

Figure S11. ^{1}H NMR spectrum of (4*RS*,5*RS*)-3-(4-chlorophenyl)-4-nitro-5-tribromomethyl-2-isoxazoline (**3d**).

^{13}C NMR

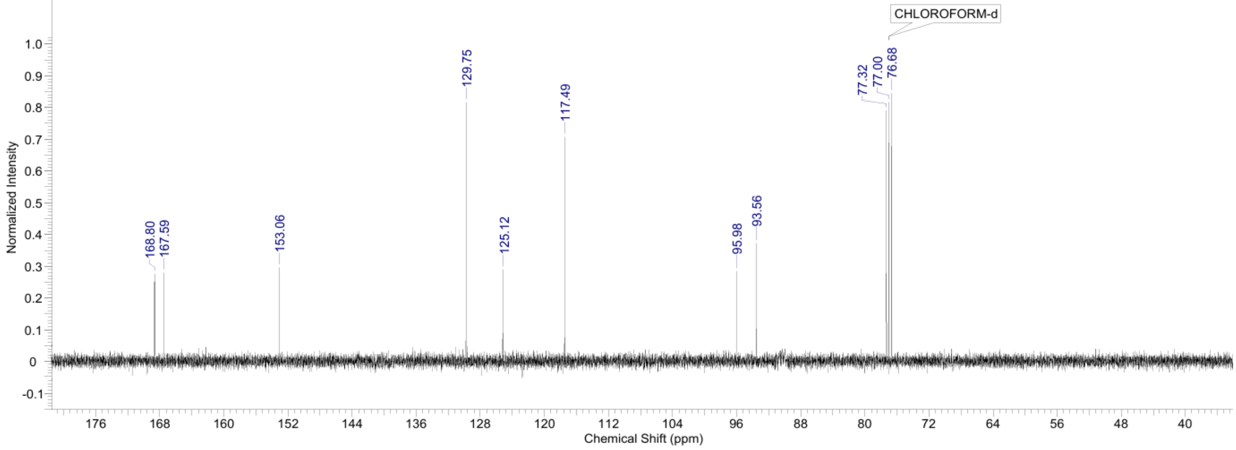

Figure S12. ^{13}C NMR spectrum of (4*RS*,5*RS*)-3-(4-chlorophenyl)-4-nitro-5-tribromomethyl-2-isoxazoline (**3d**).

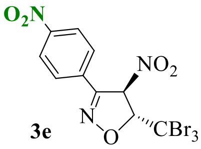

3e

(4*RS*,5*RS*)-3-(4-nitrophenyl)-4-nitro-5-tribromomethyl-2-isoxazoline (**3e**): isolation and purification process: column chromatography ($CHCl_3$: Me_2CO 8 : 2 *v*/*v*) orange oil

R_f = 0.42; UV-Vis (MeOH): λmax [nm] 255; FT-IR (ATR): υ [cm^{-1}] 1598 (>C=N− 2-isox. ring), 1552 asym. and 1344 sym. (−NO_2), 1253 (~C-O-N= 2-isox. ring), 850 (~C-Br); ^1H NMR (400 MHz, $CDCl_3$): δ [ppm] 7.92-7.86 (m, 4H); 7.04 (d, 1H, J = 4.25 Hz); 6.57 (d, 1H, J = 4.26 Hz); ^{13}C NMR (100 MHz, $CDCl_3$): δ [ppm] 156.4; 153.9; 149.5; 133.4; 130.7; 123.7; 101.0; 90.1; HR-MS (ESI−): calculated for $C_{10}H_6N_3O_5Br_3$ $[M{-}H]^-$ = 483.7779, found 483.7796

HR-MS

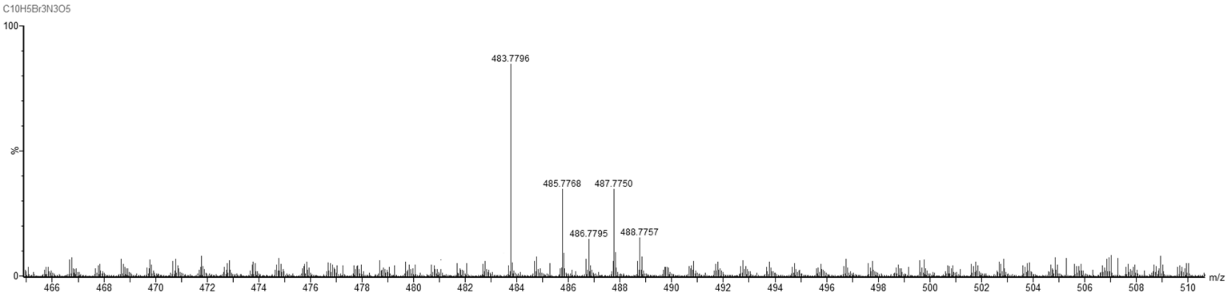

Figure S13. HR-MS spectrum of (4*RS*,5*RS*)-3-(4-nitrophenyl)-4-nitro-5-tribromomethyl-2-isoxazoline (**3e**).

^{1}H NMR

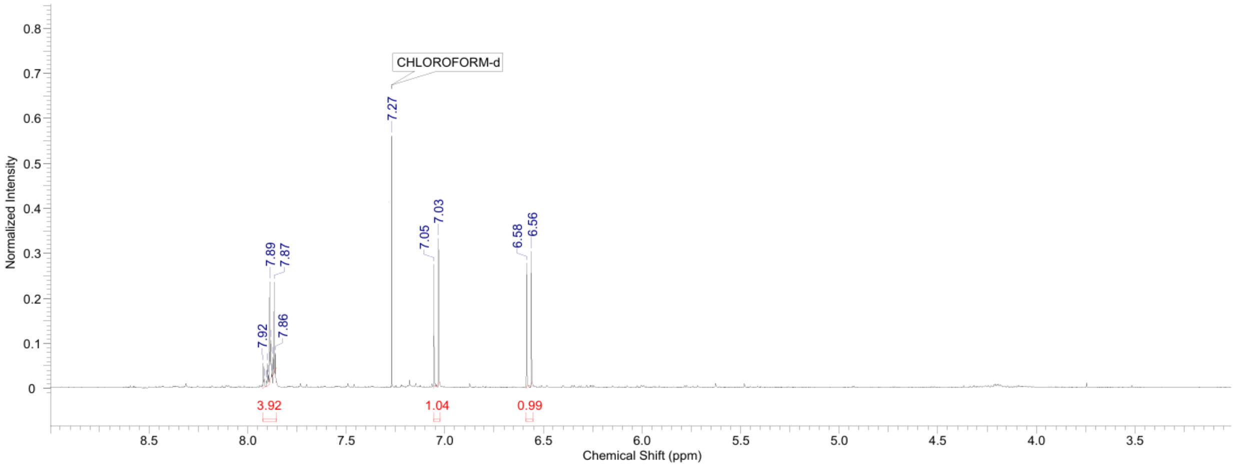

Figure S14. ^{1}H NMR spectrum of (4*RS*,5*RS*)-3-(4-nitrophenyl)-4-nitro-5-tribromomethyl-2-isoxazoline (**3e**).

^{13}C NMR

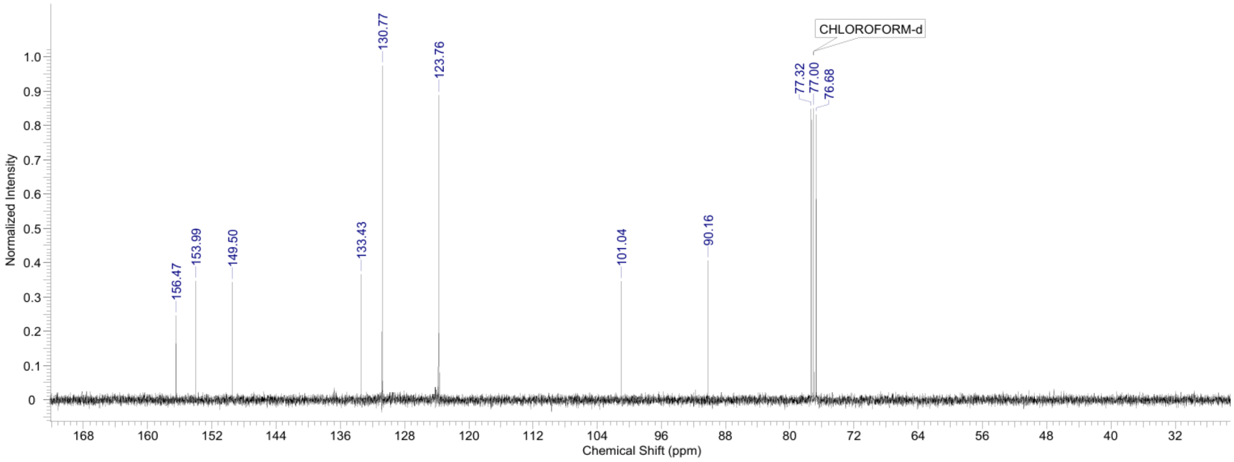

Figure S15. ^{13}C NMR spectrum of (4*RS*,5*RS*)-3-(4-nitrophenyl)-4-nitro-5-tribromomethyl-2-isoxazoline (**3e**).

Table S1. ωB97XD/6-311G(d,p) populations of the most relevant ELF valence basins of ANOs **1a-e**, in average number of electrons (e).

		**1a** (R = OMe)	**1b** (R = Me)	**1c** (R = F)	**1d** (R = Cl)	**1e** (R = NO_2)
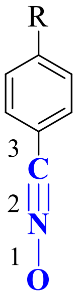	V(O1,N2)	1.55	1.53	1.55	1.53	1.57
	V(N2)	–	–	–	–	–
	V(N2,C3)	3.02	2.89	2.91	3.03	2.87
	V'(N2,C3)	3.02	3.02	3.05	2.97	3.07
	V(O1)	2.85	2.97	2.98	2.95	2.96
	V'(O1)	2.85	2.80	2.81	2.75	2.71
